# Assessment of the Relationship between Y-Balance Test and Stabilometric Parameters in Youth Footballers

**DOI:** 10.1155/2020/6968473

**Published:** 2020-11-12

**Authors:** Damian Sikora, Małgorzata Pałac, Andrzej Myśliwiec, Tomasz Wolny, Paweł Linek

**Affiliations:** ^1^Department of Kinesitherapy, The Jerzy Kukuczka Academy of Physical Education, Katowice, Poland; ^2^Musculoskeletal Diagnostic and Physiotherapy-Research Team, The Jerzy Kukuczka Academy of Physical Education, Katowice, Poland; ^3^Institute of Physiotherapy and Health Sciences, Musculoskeletal Elastography and Ultrasonography Laboratory, The Jerzy Kukuczka Academy of Physical Education, Katowice, Poland; ^4^Institute of Physiotherapy and Health Sciences, The Jerzy Kukuczka Academy of Physical Education, Katowice, Poland

## Abstract

**Objectives:**

The purpose of the study was to evaluate the correlation between dynamic test results obtained on a stabilometric platform and the results achieved on the Y-balance test (Y-BT).

**Method:**

The study group consisted of 52 adolescent athletes, aged 14 to 17 years. Each participant was evaluated in the scope of their ability to maintain dynamic balance using the Y-BT as well as via dynamic tests on the ‘Alfa' stabilometric platform. The following parameters were analysed: (a) from the Y-BT—relative reach of the right and left lower limbs in the anterior, posterolateral, and posteromedial directions, as well as the side-to side difference in relative reach for each direction and (b) from the ‘Alfa' platform—path length and time to reach the target using right and left lower limbs in the anterior and posterior directions.

**Results:**

A correlation between the results obtained on the stabilometric platform and the Y-BT was found only for the posteromedial direction. Statistical analysis demonstrated that the increased difference between the right and left lower limbs in the posteromedial test is related to an increase in time taken to reach the points located forward and to the left, and backwards and to the right, as well as an increase in the overall time required to complete the task on the stabilometric platform.

**Conclusions:**

The results from the Y-BT and stabilometric platform are weakly related in adolescents. These findings indicate that the Y-BT and stabilometric platform analyse different kinds of dynamic balance in adolescents. Thus, these tools should not be used interchangeably in clinical practice or scientific research.

## 1. Introduction

Maintenance of dynamic balance depends on proper functioning of the postural control system, which sustains the centre of pressure (COP) within the field of support [1]. In sports, the balance control process plays an important role as diverse exercise intensity must be adapted to the conditions prevailing on the pitch, as well as to various team tactics [2]. The researchers claim that obtaining a high level of skill in sports as an adult athlete depends on the correct development of motor skills at a young age [3].

Currently, the most acclaimed tool for the evaluation of dynamic balance is the stabilometric platform, the purpose of which is to record COP movements with strain gauges placed in the platform structure [4]. In addition to the common static tests (known as COP oscillation), it is also possible to perform dynamic tests that record the movement of COP during a specific motor task. Another way to assess dynamic balance is the star excursion balance test (SEBT), which is carried out by using lines placed on the floor [5–7] or by using the Y-Balance Kit. Thus, the SEBT test—using the Y-Balance Kit—is often referred to in research as the Y-balance test (Y-BT) [8–10]. The Y-BT evaluates a user's ability to maintain balance while performing movements in the anterior, posterolateral, and posteromedial directions. A wide number of applications, including many in sports and adult rehabilitation [5] as well as in the examination of children and adolescents [11, 12], have found this test useful.

It is assumed that the SEBT test, when performed on the stabilometric platform, is characterised by high reliability [13]; however, this depends on the adopted testing protocol [14]. The Y-BT includes only one type of test, which is also characterised by high reliability in adults and good reliability in adolescents [5, 15]. Considering the high costs of purchasing the stabilometric platform, it seems reasonable to ponder whether, to a certain extent, both tools may be used interchangeably. Since both tests measure dynamic balance, there should be a correlation between the two methods of measurement. Therefore, the purpose of the present study was to evaluate the correlation between the results obtained on a stabilometric platform and those achieved in the Y-BT.

## 2. Materials and Methods

### 2.1. Participants

A sample of 52 male athletes between 14 and 17 years of age were selected from a semiprofessional football club. Inclusion criteria were as follows: (a) dominant right lower leg (i.e., the right leg is preferable during kicking); (b) minimum score for each of the questions from the Oslo Sports Trauma Research Centre questionnaire (OSTRC) [16], regarding injury, illness, or other health problems during the past week prior to the study; and (c) no injuries that prevented training or competition participation for longer than one week, within a period of four months prior to the examination. Full characteristics of the participants are presented in Table 1. All participants and their parents or legal guardians received oral and written information about all procedures and gave their signed informed consent to participate. The study was designed according to the Declaration of Helsinki and was approved by the local ethical committee.

### 2.2. Y-Balance Test

**Figure 1:**
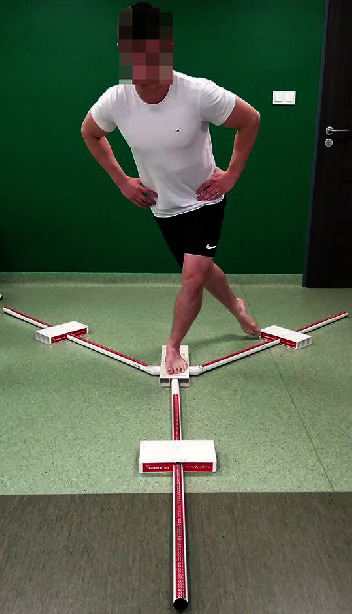
*Y*-balance test.

The examination of balance was performed using the Y-BT Kit [5]. The Y-BT Kit comprises a single central plastic plate and three attached tubes arranged in the following directions: anterior, posteromedial, and posterolateral. The participants, while standing on one leg (barefoot) in a central location on the Y-BT instrument with their hands placed on the wings of their ilium, were asked to move the pointer (the central plastic plate) as far as possible in the directions mentioned using the lower limb opposite to their support limb (Figure 1).

The whole procedure was the same as in a prior study by Lee et al. [17]. After six practice trials, the results from three subsequent test attempts in each direction were recorded, separately, in centimetres. The mean values from the three attempts for each direction were used to calculate the normalised (by length of the lower limb) percentage value of the distance obtained. Therefore, the length of the tested lower limb was also measured using a tape measure while the participant was lying down on the therapeutic table. The relative lower limb length was measured from the anterior superior iliac spine to the medial malleolus. In further analysis, the relative lower limb length was used in Equation (1) to compute the normalised percentage value of the distance obtained [15, 18].

(1) $$\begin{array}{c} \text{Normalised}\text{percentage}\text{value}=\frac{\text{the}\text{mean}\text{distance}\text{obtained}\text{in}\text{each}\text{direction}}{\text{relative}\text{length}\text{of}\text{the}\text{limb}}x100, \end{array}$$

Additionally, the relative side-to-side difference of the Y-BT was calculated using

(2) $$\begin{array}{c} \text{Side}‐\text{to}‐\text{side}\text{difference}=\left|\text{distance}\text{obtained}\text{by}\text{the}\text{right}\text{leg}–\text{distance}\text{obtained}\text{by}\text{the}\text{left}\text{leg}\right|, \end{array}$$

### 2.3. Stabilometric Platform

Dynamic tests were carried out on the ‘Alfa' (AC International East—www.acinternational-east.pl/en/alfa-2/) stabilometric platform. Each participant was in a free position on the platform, placing their bare feet parallel to one another and maintaining a distance of 10 centimetres, measured from the head of the first metatarsal bone to the centre line of the platform. The lateral ankles were on the perpendicular line dividing the platform into halves, running 15 centimetres from the rear edge of the platform, while the participants' hands were resting on the wings of the ilium. While assessing balance during the dynamic test, the participant standing on the platform moved their COP without lifting their feet from the platform, according to the instructions on the screen. The round fields on the screen were successively illuminated, and the task of the athlete was to tilt the centre of gravity towards the illuminated virtual object without using their hands or taking their feet off the ground. Each participant performed three practice trails. Then, three consecutive attempts were recorded. The study evaluated measurements of the path length of the COP movement, which is the total distance the COP travelled within 30 seconds, expressed in millimetres.

### 2.4. Statistical Analysis

Data from all participants were used to examine correlations between Y-BT (normalised values of anterior, posterolateral, and posteromedial directions) and stabilometric parameters (path length and time to reach target). Additionally, relative side-to-side differences on the Y-BT and among stabilometric parameters were correlated. Due to the lack of a normal distribution of the variables studied (confirmed by the Shapiro–Wilk test), a nonparametric Spearman rank correlation analysis was applied. Spearman's *r* was interpreted as negligible (0.00-0.10), weak (0.10-0.39), moderate (0.40-0.69), strong (0.70-0.89), or very strong (0.90-1.00) [19]. Significant differences were assumed at the level of *p* ≤ 0.05.

## 3. Results

### 3.1. Y-BT Side-to-Side Difference

The correlation analysis demonstrated that the increase in the length difference between the right and left lower limbs in the posteromedial direction of the Y-BT is weakly related to an increase in the time taken to reach the points located forward and to the left, and backwards and to the right, as well as an increased overall time required to complete the task in the stabilometric parameters (see Table 2).

### 3.2. Y-BT Reach Test

A moderate correlation was found between the normalised value in the posteromedial direction by both limbs on the Y-BT and the time needed to complete a task on the stabilometric platform. The higher values for the posteromedial distance in the Y-BT were correlated with lower values for the time needed to complete a task on the stabilometric platform. Additionally, the higher values of posteromedial distance in the Y-BT were weakly correlated with a reduction in the time required to reach a target on the left side (anterior and posterior) of a platform test. Likewise, a weak relationship was found between the distance achieved for both limbs in the anterior direction (Y-BT) and the time necessary to reach the target located on the right anterior side (see Table 3).

## 4. Discussion

To date, the correlation between results from two different tools (Y-BT and stabilometric platform) assessing dynamic balance has never been studied in adolescents. This study demonstrates that there are relationships between the dynamic tests performed on the stabilometric platform and the Y-BT. However, the relationships were mostly weak (*r* < 0.4). Only a moderate correlation was demonstrated between the time needed to complete the task on the stabilometric platform and the normalised distance value obtained on the Y-BT in the posteromedial direction. This means that study participants who achieved better results on the Y-BT for the posteromedial direction required less time to complete the task on the stabilometric platform.

In the absence of other studies on the degree of relationship between the Y-BT and the dynamic tests on the stabilometric platform, it is impossible to compare the obtained results with the results from similar research on adolescents. In turn, there are some studies analysing the relationship between the Y-BT (such versions with adhesive lines on the floor) and the Biodex platform in adults [20, 21]. Almeida et al. [20] have shown no correlation between the results obtained from both applied research tools (Y-BT and Biodex), explaining that the lack of correlation was due to various factors affecting the assessment of balance, and suggesting a higher accuracy of the tests performed on the Biodex platform. Glave et al. [21] have observed a quite surprising negative correlation between the Y-BT and the Biodex platform—this means that participants obtaining a better result using the Y-BT obtained a worse result on the Biodex platform. The current study also demonstrated similar results as mentioned in papers on adult subjects, as (a) we mostly found no or weak correlations between the Y-BT and stabilometric platform and (b) we also detected an unexpected relationship—participants obtaining better results using the Y-BT in the anterior direction (for both legs) required longer times to reach the target in the rightanterior direction on the stabilometric platform.

The level of correlation obtained in the present study and the resulting coefficient of determination allow us to establish that, at the most optimal assumption, the time achieved on the stabilometric platform (i.e., the time required to reach the target) is only approximately 28% explained by the results obtained on the Y-BT (and only in terms of the posteromedial direction). This observation, together with an unexpected correlation of the anterior direction in the Y-BT and the time necessary to reach the target (right-anterior) on the platform, confirm the suggestions of other researchers [20, 21] that both tests measure a different kind of dynamic balance and allow us to evaluate other elements of dynamic balance. Thus, these tools are not interchangeable in adults nor in this study on adolescents.

Hence, the analysis of dynamic balance in adolescent athletes must be closely linked with the tool and study design since high scores on one test (e.g., on the platform) will not substantiate high performance on another (e.g., on the Y-BT) that evaluates underlying motor ability. In the literature, dynamic balance is defined as the ability to maintain stability during complex motor tasks, which is essential for the activities of everyday life and for sports [22, 23]. Although this study included the stabilometric platform test, which slightly differed from the Y-BT, both tests should evaluate the character of movement and in some way be related, assuming that they concern the same phenomenon (e.g., dynamic balance). However, the lack of association between Y-BT and stabilometric parameters clearly indicates the need for further research to define the specific types of balance [21] that are evaluated by each tool. Each method should be specifically evaluated for reliability and its potential should be determined for possible use in scientific research (e.g., to estimate the possibility of the occurrence of injury or to determine current level of training).

## 5. Conclusions

The results from the Y-BT and stabilometric platform are weakly related in adolescents. Correlation of the Y-BT test results with the results obtained on the stabilometric platform indicates that the Y-BT analyses a different kind of dynamic balance. Thus, these tools should not be interchangeably used in clinical practice or scientific research.

## Figures and Tables

**Table 1:** Basic data of participants—mean and standard deviation (SD).

Characteristic (*n* = 52)	
Age (yr)	15.5 (1.0)
Weight (kg)	59.9 (10.3)
Height (cm)	174.2 (7.7)
BMI (kg/m^2^)	19.7 (2.4)
Sports practice (yr)^a^	7.7 (1.0)
Right dominant leg^b^	100%
OSTRC (%)^c^	
Full participation without any problems	100%
No training reduction	100%
No performance reduction	100%
No symptoms	100%

^a^For how many years soccer has been practice prior to the study? ^b^Which leg do you prefer when playing football? ^c^ % number of participant's declaration; OSTRC: Oslo Sports Trauma Research Centre questionnaire; BMI: body mass index.

**Table 2:** Relationship between relative side-to-side difference of the Y test and stabilometric platform results.

Y test	A direction	PL direction	PM direction
Alpha platf			
Path length	R = −0.10	R = 0.03	R = −0.01
Time to reach right—A	R = 0.02	R = 0.04	R = 0.07
Time to reach left—A	R = −0.01	R = 0.07	*R* = 0.30^∗^
Time to reach left—P	R = 0.06	R = −0.01	*R* = 0.23
Time to reach right—P	R = −0.02	R = 0.02	*R* = 0.30^∗^
Time	*R* = −0.07	*R* = 0.04	*R* = 0.27^∗^

^∗^Significant correlation *p* ≤ 0.05; A: anterior; P: posterior; PL: posterolateral; PM: posteromedial.

**Table 3:** Relationship between Y test reach and stabilometric platform results.

Y test	Right leg			Left leg		
Alpha platf.	Anterior direction	PL direction	PM direction	Anterior direction	PL	PM direction
Path length	*R* = 0.13	*R* = −0.12	*R* = −0.13	*R* = 0.13	*R* = −0.14	*R* = −0.10
Time to reach the target right—anterior	*R* = 0.34^∗^	*R* = −0.08	*R* = −0.24	*R* = 0.35^∗^	*R* = −0.11	*R* = −0.25
Time to reach the target left—anterior	*R* = 0.10	*R* = −0.18	*R* = −0.29^∗^	*R* = 0.08	*R* = −0.18	*R* = −0.35^∗^
Time to reach the target left—posterior	*R* = 0.16	*R* = −0.28^∗^	*R* = −0.24	*R* = 0.03	*R* = −0.22	*R* = −0.41^∗^
Time to reach the target right—posterior	*R* = 0.04	*R* = 0.23	*R* = −0.02	*R* = 0.04	*R* = 0.03	*R* = −0.09
Time	*R* = 0.13	*R* = −0.19	*R* = −0.41^∗^	*R* = 0.12	*R* = −0.30^∗^	*R* = −0.53^∗^

^∗^Significant correlation *p* ≤ 0.05; PL: posterolateral; PM: posteromedial.

## Data Availability

Data is available on request.
